# The Darkest Place Is under the Light House

**DOI:** 10.3201/eid1304.000000

**Published:** 2007-04

**Authors:** Polyxeni Potter

**Affiliations:** * Centers for Disease Control and Prevention, Atlanta, Georgia, USA

**Keywords:** Utagawa Hiroshige, Ichiryūsai Hiroshige, Ryusai, Andō Hiroshige, Plum Garden at Kameido, Floating world, Japanese woodblock prints, zoonotic and vectorborne interactions, ukiyo-e, One Hundred Views of Edo, art and science, art commentary, about the cover

**Figure:**
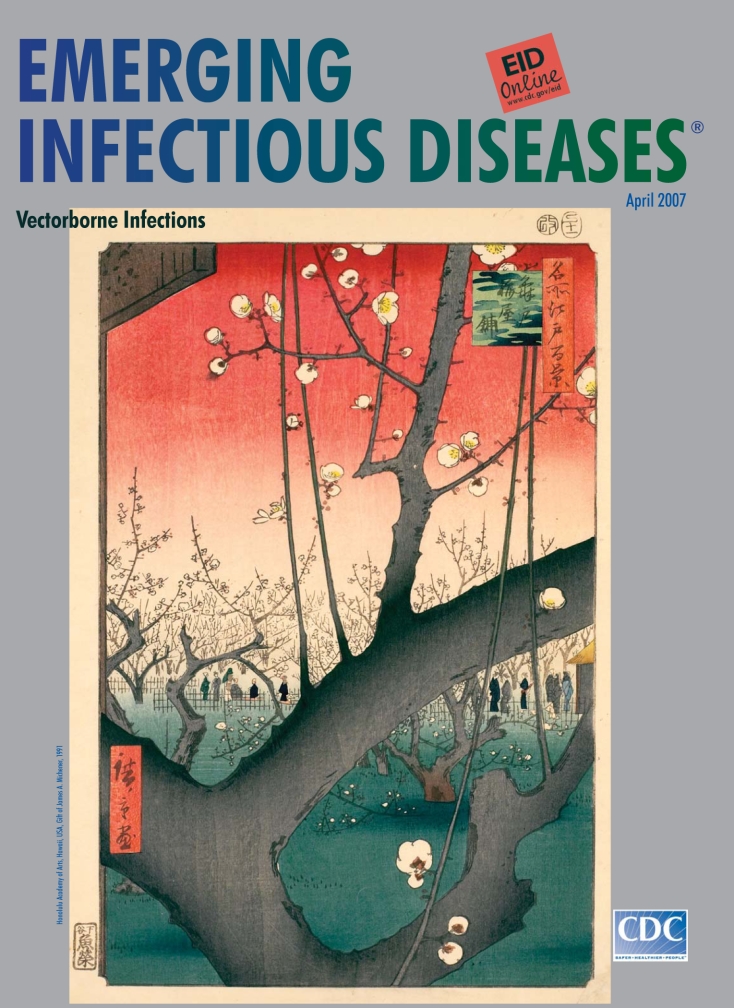
**Utagawa Hiroshige (1797–1858). Plum Garden at Kameido (1857).** From the series One Hundred Views of Edo. Color woodblock print (37.7 cm × 26.5 cm). Honolulu Academy of Arts, Hawaii, USA, Gift of James A. Michener, 1991 (24103)

– Japanese proverb

“Hiroshige’s death cannot be too much deplored,” read the note next to his name on a list of the famous who perished in the 1858 cholera epidemic (*1*). Utagawa Hiroshige himself, before his death at age 62, is said to have written in verse, “I leave my brush at Azuma and go on the journey to the Holy West to view the famous scenery there” (*2*). Metaphorical meaning and questionable authenticity aside, when interpreted in Western terms, the farewell becomes prophetic since the artist, long overlooked in his native Japan, was discovered and brought to light in the West.

The low cost of production made woodblock prints, Hiroshige’s main medium, widely accessible—a print sold for the price of a bowl of noodles (*3*). The subjects, eloquent snapshots of the provinces, remained unknown to the cultured class in Japan, until his popularity rose in Europe and the United States. How the prints first found their way to Western artists, who appreciated and imitated them is legend. James McNeill Whistler (1834–1903), who was very influential in introducing Japanese art to England, might have seen his first Hiroshige at a rundown Chinese tea house near London Bridge or on the wrapper on a pound of tea (*2*).

Van Gogh so admired Japanese prints that he copied some, among them Plum Garden at Kameido, on this issue’s cover. “I envy the Japanese artists,” he wrote, “for the incredible neat clarity which all their works have” (*4*). Hiroshige’s influence was greatest on the impressionists (Claude Monet, Pierre August Renoir, Mary Cassatt) and postimpressionists (Paul Cézanne, Henri de Toulouse-Lautrec, Paul Gauguin). His landscapes, “simple as breathing,” “easy as buttoning one’s waist-coat,” influenced Western painting away from representation toward light, color, and emotion (*4*).

Hiroshige’s biography is pieced together from anecdotes as there are few authentic records of his life. He was born Andō Tokutarō in Edo, present-day Tokyo, a precocious child with an eye for the unusual and the detailed. Orphaned in his early teens, he managed to receive art instruction, first from amateur painter Okajima Rinsai, a friend and neighbor, and at age 15, from the art establishment. In the short course of a year, he was admitted to the Utagawa School as designer of color prints. According to Japanese custom, an accomplished apprentice was given a name that generally incorporated part of the master’s name. Apprentice of Toyo**hiro**, Andō was named **Hiro**shige. The diploma, in Toyohiro’s own writing, read Utagawa Hiroshige. The artist later also used the names Ichiryūsai and Ryusai.

Hiroshige’s father, Gen’emon Andō, a hereditary retainer of the shōgun, was a fireman, and when he died, Hiroshige kept his modest post, eking out a living until he could relinquish the post to another member of the family and devote himself to art. Then he set off to see and draw the provinces. A wanderer and bon vivant, he lingered on the road, pausing to observe and sketch, mixing with country folk, dining at local eateries. His discriminating tastes and humorous accounts of people and places, recorded in his diaries, were largely lost in the 1896 earthquake, but his absorption with natural beauty, which he held “exquisite, beyond capability of describing with brush” (*1*), survived in his landscapes.

Legend has it that seeing the work of master printmaker Katsushika Hokusai (1760–1849) inspired Hiroshige to become a *ukiyo*-*e* artist, to create images of the “floating world.” These images, drawn from the transient world of actors and others in Edo’s theater district, expanded to encompass scenes of nature and eventually the life of the common people: “Living only for the moment, turning our full attention to the pleasures of the moon, the snow, the cherry blossoms, and the maple leaves; singing songs, drinking wine, diverting ourselves in just floating, floating…like a gourd floating along with the river current; this is the floating world” (*5*).

Hiroshige formed his own interpretation of the floating world, which he summed up in the inscription on The Hundred Views of Mount Fuji: “…the old man [Hokusai] had drawn grasses, trees, birds, animals, and other things in his usual talented brush….his work focused upon making things interesting….I simply reproduce sketches of what I had seen before my eyes” (*6*). But true to life as Hiroshige was, he captured the pathos, not the details, favoring white spaces, flattened forms, organic scenes in brilliant color. Like van Gogh, he tried to draw not “a *hand*, but the gesture, not a mathematically correct head, but the general expression….In short, *life*” (*7*).

Hiroshige was very prolific. He created thousands of images of his beloved Edo and surrounding provinces—bridges, roads, temples—under all manner of weather, day and night. He named them all personally, as he also had a talent for verse, which he dispersed generously and with wit. His prints were copied and reprinted freely.

Plum Garden at Kameido, in a series created just a year before his death, shows the master’s unparalleled facility with Japanese topography. This close-up of a plum tree, thick trunk framing the famed gardens, young sprouts shooting out the edge, blossoms placed seductively against a sky flushed pink, is an essay in perspective. The tree trunk, a main attraction, is what we are less likely to see, its spare immediacy too close to the lens. Hiroshige wants us to venture past it into the spring extravaganza beyond.

Perspective, a Western influence, graces many of Hiroshige’s later works, which having excelled in capturing life as he saw it, now explored its depth. Master of illusion, he brought what he saw into focus, knowing full well that the scene was but a composition of life elements, not life itself.

Hiroshige’s dilemma with perspective is not unlike the scientist’s, who also draws selected objects closer for a better look. But magnification and clarity are no guarantee of true perspective in the laboratory any more than in art. Out in the open, under the proverbial lighthouse, lies always the risk of missing the obvious in close and plain view. And despite the science frame, zoonotic and vectorborne interactions and connections within the natural environment, like the strolling visitors in the garden at Kameido, can easily be overlooked.
